# Early MinION^™^ nanopore single-molecule sequencing technology enables the characterization of hepatitis B virus genetic complexity in clinical samples

**DOI:** 10.1371/journal.pone.0194366

**Published:** 2018-03-22

**Authors:** Virginie Sauvage, Laure Boizeau, Daniel Candotti, Mathias Vandenbogaert, Annabelle Servant-Delmas, Valérie Caro, Syria Laperche

**Affiliations:** 1 Institut National de la Transfusion Sanguine (INTS), Département D’études des Agents Transmissibles par le Sang, Centre National de Référence Risques Infectieux Transfusionnels, Paris, France; 2 Institut Pasteur, Genotyping of Pathogens Pole, Laboratory for Urgent Response to Biological Threats, Environment and Infectious Risks, Paris, France; Oklahoma State University, UNITED STATES

## Abstract

Until recently, the method of choice to characterize viral diversity consisted in cloning PCR amplicons of full-length viral genomes and Sanger-sequencing of multiple clones. However, this is extremely laborious, time-consuming, and low-throughput. Next generation short-read sequencing appears also limited by its inability to directly sequence full-length viral genomes. The MinION^™^ device recently developed by Oxford Nanopore Technologies can be a promising alternative by applying long-read single-molecule sequencing directly to the overall amplified products generated in a PCR reaction. This new technology was evaluated by using hepatitis B virus (HBV) as a model. Several previously characterized HBV-infected clinical samples were investigated including recombinant virus, variants that harbored deletions and mixed population. Original MinION device was able to generate individual complete 3,200-nt HBV genome sequences and to identify recombinant variants. MinION was particularly efficient in detecting HBV genomes with multiple large in-frame deletions and spliced variants concomitantly with non-deleted parental genomes. However, an average-12% sequencing error rate per individual reads associated to a low throughput challenged single-nucleotide resolution, polymorphism calling and phasing mutations directly from the sequencing reads. Despite this high error rate, the pairwise identity of MinION HBV consensus genome was consistent with Sanger sequencing method. MinION being under continuous development, further studies are needed to evaluate its potential use for viral infection characterization.

## Introduction

The genetic complexity of a viral population in an infected host depends on several complex molecular mechanisms involving nucleotide substitutions, insertion/deletion events, inter- and intra-genotype recombination, and re-assortment events. The resulting viral genetic diversity has significant implications in epidemiology, molecular and serologic diagnosis, pathogenesis, and therapeutic management [1]. Thus, new molecular methods are continuously developed to improve the characterization of this intra-host viral diversity. Common approaches used Sanger sequencing of amplicons of various sizes covering several sub-genomic regions of interest of the viral genome but rarely the complete genome. Sequencing of a mixture of amplicons with the Sanger method yields a consensus sequence corresponding to nucleotides present at least in 10–20% of the overall population, but no sequence of individual virions. In addition, consensus approach does not clearly distinguish between true inter-virion genetic recombination and dual infections in infected individuals [2]. Until recently, the method of choice to analyze individual complete viral genomes from a clinical sample was cloning full-length PCR amplicons and Sanger-sequencing of multiple clones. However, this is extremely laborious, time-consuming, and low-throughput. The development of second-generation sequencing methods allowed in depth characterization of viral genomic variability by simultaneously providing large amounts (thousands to millions) of individual sequences from the same amplicon or clone. This large scale sequencing procedure allows the detection of minor variants that the standard Sanger method might miss. However, the major intrinsic limit of the second-generation sequencing systems remains the size limit of the individual sequences obtained (~700 nt) [3]. Thus, a complete viral genome sequence cannot be obtained in a single read impairing linking distant mutations in an individual genome, and possibly the clear identification of viral recombinants. However, the recently developed single-molecule third-generation sequencing systems provide a promising alternative in sequencing amplified full-length single viral genomes.

New generation of sequencing technologies such as PacBio RS II and the Sequel instruments from Pacific Biosciences, have been developed to produce long sequence reads up to 60 kb in length [4] providing the opportunity to detect the genetic diversity of a viral population at the quasispecies level without the need of an assembly step of short sequence fragments [5]. Recently, Oxford Nanopore Technologies (ONT) developed the MinION^™^ device based on modulations of ionic current as a single-stranded DNA molecule passes through a nanopore. These ionic current changes are dependent on the physical properties of the nucleic acid bases and allow determination of the nucleotide sequence [6]. Compared to other available devices, the MinION^™^ sequencer presented a low capital cost, was miniature (3.5 × 10.5 cm), portable and directly connected to a computer to perform data acquisition and analyses in real time. These features made the MinION instrument a new attractive tool with potential new opportunities and benefits for management of viral infections. In 2014, ONT opened the MinION Access Program (MAP) framework to approximately 1,000 users [6] to assess potential applications, performance and limitations of the device for their own research. As a selected laboratory, we aimed to investigate the ability of the MinION nanopore long-read single-molecule sequencing system as an alternative to traditional cloning and Sanger sequencing procedures for studying the viral population genetic diversity using individuals infected with the hepatitis B virus (HBV) as a model.

HBV remains a global public health issue despite the availability of a vaccine and antiviral treatments. The approximately 3,200 nucleotides (nt) long, relaxed circular, partially-double stranded HBV DNA genome has a compact coding structure containing four overlapping reading frames encoding the reverse transcriptase/DNA polymerase (P), core (C), envelope (S), and X proteins. The viral genome replication involves a polyadenylated pre-genomic RNA that is reverse transcribed into cDNA by the viral reverse transcriptase (RT). The lack of proofreading activity of the RT (estimated error rate ranging between 1 per 10^4^ and 1 per 10^7^ nucleotides according to the studies of Gunther *et al*. [7] and Nowak *et al*. [8]) associated with a rapid viral turnover in infected cells results in the emergence of significant genetic diversity among HBV isolates and a variable complex HBV quasi-species distribution within hosts during the natural course of infection. Based on this genetic diversity, eight confirmed (A-H) and two tentative (I and J) genotypes have so far been described based on an internucleotide divergence greater than 7.5% across the complete genome [9]. Subgenotypes were further defined by an intra-genotypic nucleotide divergence >4%. These genotypes/subgenotypes differ in their geographic distributions, transmission routes, disease progression, response to antiviral treatment, and clinical outcomes [9,10]. Furthermore, co-infections potentially leading to intergenotype recombinations have been described worldwide. Intergenotype recombination constitutes an important element of HBV genetic variability with potential clinical implications [2, 11].

As part of the initial MAP evaluation, long read nanopore sequencing performance was evaluated using previously characterized HBV strain samples including either recombinant viruses, mixed populations of wild-type viruses and variants with major deletions, and mixed populations of single-nucleotide variants.

## Materials and methods

### Samples

Three archived plasmas from the HBV-infected blood donor samples collection (DC 2016–2842) of the National Reference Center Infectious risks in blood transfusion, INTS, were included in the study. As shown in Table 1, sample B5584 was infected with a 3,209-nt length genotype D1/E recombinant virus. Sample B6260 contained a complete 3,179-nt genotype D2 virus and a related 3,032-nt variant that harbored 123-nt and 24-nt deletions in the Pre-S1 and S regions, respectively. Sample B6505 contained a mixed population of 3,220-nt genotype A2 variants identified by sequence ambiguities within the “a-determinant” of the S gene at positions 501 (C/A) and 506 (A/T). These C501A and A506T substitutions leading to sThr116Asn and sThr118Ser mutations that have been previously associated with vaccine escape and diagnostic failure [12].

**Table 1:** HBV genetic diversity in three infected individuals characterized by sequencing whole genome PCR products using three different methods.

Sample	HBV genotype	Direct Sanger sequencing	Molecular cloning	Direct MinION sequencing
B5584	D1/E	Max. sequence length: 3,209-nt	Max. sequence length: 3,209-nt	Max. sequence length: 3,201 nt
		D1/E recombinant sequence	Individual D1/E recombinant sequences	Individual D1/E recombinant sequences
		Single nucleotide ambiguities across whole genome	D1 and E parental sequences not detected	D1 and E parental sequences not detected
				Single nucleotide ambiguities across whole genome*
				1,229-nt deletion (pos. 2,471–489)—putative spliced variant
B6505	A2	Max. sequence length: 3,220-nt	Not done	Max. sequence length: 3,217-nt
		Mixed population of wild-type and single nucleotide mutant S sequences		Mixed population of wild-type and single nucleotide mutant S sequences
				Evaluation of mutation association patterns
				1,049-nt deletion (pos. 2,447–282)—putative spliced variant
				96-nt deletion (pos. 2,131–2,226)
B6260	D2	Overlapping sequence signals suggesting at least 2 sequences coexisting	Individual wild-type sequences (3,179-nt)	Max. sequence length: 3,015-nt
		Single nucleotide ambiguities across whole genome	Individual sequence (3,032-nt) with 123-nt (pos. 2,929–3,051) and 24-nt (pos. 501–524) deletions	Individual wild-type sequences
				Individual sequences with 123-nt (pos. 2,929–3,051) deletion
				Individual sequences with 123-nt (pos. 2,929–3,051) and 24-nt (pos. 501–524) deletions
				Single nucleotide ambiguities across whole genome*

* Single nucleotide sequence ambiguities were not investigated further.

Abbreviations: gt, genotype; nt, nucleotide; pos, position.

HBV genotyping was performed by direct PCR product sequencing and phylogenetic analysis of partial sequences of the S gene as previously described [13]. HBV DNA loads of 6.45, 2.88, and 4.86 log IU/mL were observed in samples B5584, B6260 and B6505, respectively, by using the High Pure System/COBAS TaqMan HBV assay (Roche Diagnostics, Meylan, France).

### Full-length HBV genome amplification, cloning and Sanger sequencing

Full length HBV-DNA was amplified according to the protocol described by Gunther *et al*. [14]. Amplicons ~3.2 kb in length were gel-purified by using the QIAquick Gel Extraction Kit (Qiagen, Courtaboeuf, France) and sequences were determined by the Sanger method. In addition, full-length PCR products of samples B5584 and B6260 were cloned into pCR-XL-TOPO^®^ vector (Life Techologies, Villebon sur Yvette, France) according to the manufacturer’s instructions. For each sample, two to five clones were sequenced.

### MinION^™^ library preparation

MinION^™^ sequencing libraries were prepared directly from the total products of HBV full-length genome PCR reactions after purification with the MinElute PCR Purification kit (Qiagen) and from cloned full-length HBV genomes after enzymatic linearization of the plasmid vector; linearized plasmids were purified with Agencourt AMPure XP beads (Beckman-Coulter, Villepinte, France). Briefly, the protocol for preparing a nanopore sequencing with SQK-MAP004 is as follows: 500 ng to 1μg of PCR products or cloned HBV DNA were diluted to 80μL using nuclease-free water and end-repaired using the NEBNext End Repair Module (New England Biolabs, Evry, France). The end-repaired products were then purified using 1.0 x volume of AMPure XP beads (Beckman-Coulter) and eluted in 25μL of nuclease-free. The End-repaired step was followed by a dA-tailing step of dsDNA product using the NEBNext dA-Tailing Module (New England Biolabs), prior to ligation of ONT-specific adapters with Blunt/TA Ligase Master Mix (New England Biolabs). All purifications were performed with Agencourt AMPure XP beads. Dynabeads His-Tag Isolation & Pulldown (Life Technologies) was used to elute the library in 25μL elution buffer provided by ONT.

Following the continuous development of the MinION^™^ system during the study, libraries were also prepared using the version SQK-MAP005. The volume of HP adapters ligated to dA-tailed product was then increased to 10 μL and the 2X SQK-MAP004 wash buffer was replaced by the 2X SQK-MAP005 Bead Binding Buffer.

### MinION^™^ runs

Prior to sequencing, the MinION^™^ device was connected to a computer using a USB 3.0 cable and port. A MinION^™^ R7.3 flow-cell was inserted and a quality control was run using the MinKNOW control software to assess pore activity (quality and number of functional pores). The MinION^™^ flow-cell was then loaded with 6 μL of the library mixed with 140 μL of 1X SQK-MAP004 EP buffer, or 75 μL 1X SQK-MAP005 Running Buffer + 66 μL nuclease free water, and 4 μL of SQK-MAP004 Fuel Mix or 3 μL SQK-MAP005 new Fuel Mix. All sequencing runs were performed for 18 hours on average using the standard 48-hour sequencing protocol (MAP_48H_Sequencing_Run) without reloading of the library during the run. Each flow-cell was used to run a single sample.

### MinION^™^ raw sequencing data analysis

Each read generated during a sequencing run is stored in an individual fast5 file. The raw MinION^™^ sequence data are uploaded in real-time for base calling with the software Metrichor^™^ Agent using the R7 2D Basecalling workflow (v 2.26.1 to v 2.38.3). MinION^™^ sequencing generates up to three types of read for each DNA molecule: “Template” corresponding to the forward strand, “Complement” corresponding to the reverse strand and “2D read” corresponding to a consensus sequence of the Template and Complement reads. Metrichor Agent returns files into two folders: “pass” and “fail”. “pass” contains only fast5 files where 2D base-calling was successful and the mean quality of the 2D read is ≥ 9 Phred score.

The MinION^™^ “pass”-labeled reads (encompassing together template, complement and 2D reads) from each sample were then aligned against HBV reference genomes, plus the 3.56 kb sequence of the lambda phage spike-in. LAST v475 [15] was used to acquire the final mapping results, with the following default parameters: a match score (r) of 1, a mismatch penalty of 1, and a gap open (a)/extend (b) penalty both of 1.

The alignment identity was determined as the number of matching bases divided by the total number of bases in the alignment, defined as the sum of the number of matches, mismatches, insertions and deletions. This measure is also referred to as alignment accuracy. This approach is similar to calculation of the ‘total percent error’ defined in Ip *et al*. [6], and is defined as the percentage of a read that is inaccurate due to miscalled bases, inserted bases, and deleted bases that are missing from the read but present in the reference sequence.

To determine the “total error rate” in a sequence read generated by the MinION^™^ sequencer, the number of mismatch positions was counted in the gapped alignment of a read to a reference sequence, thus providing a measure of nucleotide substitution, insertion and deletion errors. The “total error rate” was expressed as a percentage of the length of reference aligned against. In order to reduce this “total error rate”, reads were corrected using NanoCorrect, as described by Loman *et al*. [16]. A single round of correction was carried out for each individual sample.

### Genetic recombination analysis

Breakpoint positions for genomic recombination in HBV whole genome sequences were determined by using the jumping profile hidden Markov model (jpHMM-HBV) available at the Department of Bioinformatics of the Göttingen University, Germany (http://jphmm.gobics.de/submission_hbv) [17]. Sequence position numbers are provided according to an HBV genotype A reference sequence (Genbank AM282986).

### Genetic and structural variants identification

Integrative Genomics Viewer (IGV) browser (version 2.3.89) was used to identify deletions/insertions, single nucleotide variants and mutations. [18]. To phase the mutations, the reads completely covering genomic regions with direct clinical relevance were extracted.

### Accession numbers

All MinION nanopore data supporting the results of this article have been submitted to the SRA NCBI archive with the study number SRP129826 [accession number of each sample: B5584 _HBV genotype D1/E recombinant virus_ Full genome_Clone (SAMN08367587), B5584 _HBV genotype D1/E recombinant virus_ Full genome_ PCR product (SAMN08367588), B6260_ HBV genotype D2_Full genome_Clone (SAMN08367589), B6260_ HBV genotype D2_Full genome_ PCR product (SAMN08367590), B6505_ HBV genotype A2 _ Full genome_ PCR product (SAMN08367591)] and BioProject number PRJNA430224.

The Ion Torrent dataset was first preprocessed to remove duplicates and sequences of low quality (minimum quality score of 18), followed by further screening and removal of human-derived sequences using DeconSeq version 0.4.3, removing reads with a minimum overlap of 95% and >94% similarity to the human reference sequences. The corresponding Ion Torrent data are available on the SRA NCBI archive (BioSample number SAMN08376066) with the study number SRP130150 and BioProject number PRJNA430533.

## Results

### Total error rate evaluation of individual HBV reads generated by the MinION^™^ R7.3 flow cell

To evaluate the quality of reads generated by the MinION^™^ technology, two clones derived from samples B5584 (B5584c) and B6260 (B6260c) were sequenced with two distinct flow cells. After base-calling in Metrichor, B5584c and B6260c generated 1,875 and 4,962 pass reads over 7,656 and 25,562 total reads, respectively (Table 2). In both experiments, reads had a length spanning from 226 bp to 20,498 bp with a mean of ~5,600 Kb that includes sequences of HBV and vector. Available aligners LAST v475 [15], BLASR [19] and LASTZ v 1.02.00 [20] were tested to align nanopore reads to their respective Sanger sequence references including HBV and vector sequences. LAST was the read aligner generating the largest number of aligned reads, 88.1%-89.1%, with a greater alignment length average, 3,273–3,388 nt, and a mean alignment identity ranging from 72.8% to 74.6% (S1 Table). The reads that did not align to the reference sequences were mainly identified as lambda-like cloning vector sequences.

**Table 2:** LAST read mapping statistics for HBV sequencing runs on the MinION^™^ device.

				LAST^a^									
	Total reads	Pass reads	Sanger sequence length (nt)	No of mapped reads	Mean Alignt read length (nt)	Max Alignt read length (nt)	Mean Alignt Identity (min-max)	Mean Miscall rate	Mean Insertion rate	Mean Deletion rate	Mean total error rate	No of reads covering ≥ 95% of the Sanger sequence	Mean total error rate (sequences covering ≥ 95% of the Sanger sequence)
**B5584c**	**7,656**	**1,875**	3,209	**1,618**	3,060	3,209	74.8%	12.25%	3.8%	10.2%	26.25%	**1,439**	25.6%
							(59.4–91.6)	(1.9–23.1)	(0.4–11.2)	(1.9–25.9)	(8.5–42.2)		(8.5–41.3)
**B6260c**	**25,562**	**4,962**	3,032	**4,190**	2,848	3,032	73.6%	13.0%	3.65%	10.8%	27.4%	**3,545**	27.05%
							(58.9–93.6)	(0.9–26.8)	(0.0–12.1)	(2.7–26.7)	(6.4–43.2)		(8.4–43.1)

^a^ version 475

No: numberAlignment length values are rounded to the nearest whole number.

Reads corresponding to the full-length HBV DNA insert were then analyzed to evaluate the rates of three potential error types: substitution, insertion and deletion. The results showed similar mean rates of both substitution (12.5% and 13%), insertion (3.8% and 3.65%) and deletion (10.2% and 10.8%) for the two data sets obtained (Table 2). A total mean error rate of 26.25% (range: 8.5–42.2%) and 27.4% (range: 6.4–43.2%) was observed for B5584c and B6260c, respectively. This high total error rate in MinION^™^ reads appeared independent of the sequence length (S1A Fig) and the sequencing run time (S1B Fig). Furthermore, similar mean total error rates were observed when considering only reads covering ≥95% of the HBV genome (Table 2).

The NanoCorrect error correction algorithm was then applied on the pass reads (Table 3). When considering only the corrected pass reads spanning at least 95% of the viral genomes (72% and 57% of individual corrected reads for B5584c and B6260c, respectively), mean rates of substitution, insertion and deletion dropped to ~5%, ~2% and ~4.5%, respectively. Error correction of the MinION^™^ reads reduced the total error rate to ~12%. MinION^™^ derived consensus sequences showed >99% nucleotide identity across the genome with the corresponding Sanger sequences.

**Table 3:** Read mapping statistics for HBV sequencing runs after error correction using NanoCorrect.

			NanoCorrect + LAST									
	Pass reads	Sanger sequence length (nt)	No of corrected Pass reads	No of mapped reads	Mean Alignt read length (nt)	Max Alignt read length (nt)	No of reads covering ≥ 95% of the Sanger sequence	Mean Alignt Identity (min-max) (sequences covering ≥ 95% of Sanger)	Mean Miscall rate (sequences covering ≥ 95% of Sanger)	Mean insertion rate (sequences covering ≥ 95% of Sanger)	Mean Deletion rate (sequences covering ≥ 95% of Sanger)	Mean total error rate (sequences covering ≥ 95% of Sanger)
**B5584**	**1,875**	3,209	**1,673**	**1,485**	2,892	3,209	**1,065**	86.7%	5.2%	1.8%	4.6%	11.6%
							(72%)*	(60.2–99.0)	(0.0–15.7)	(0.0–7.9)	(1.0–14.0)	(1.1–32.3)
**B6260c**	**4,962**	3,032	**3,975**	**3,509**	2,555	3,032	**1,993**	88.2%	5.6%	2.1%	4.5%	12.3%
							(57%)*	(67.3–98.7)	(0.0–19.5)	(0.0–8.6)	(1.1–12.2)	(1.4–34.0)

*Percentage calculated against the number of corrected and mapped reads

No: Number

Alignment length values are rounded to the nearest whole number.

### MinION^™^ sequencing of a HBV recombinant strain

Nanopore sequencing of amplified DNA from sample B5584 produced 4,893 sequence reads from 193 of 512 flow cell channels including 472 corrected pass reads out of 735 initial pass reads (15%). All 472 corrected pass reads aligned to the HBV consensus sequence previously obtained with the Sanger method (Table 1). These individual corrected reads were 2,432 nt long on average (range: 50–3,201 nt), including 213 reads that cover ≥ 95% of the HBV genome with an average percent identity of 87% (range: 69–98%) with the Sanger sequence. As previously observed with clones, the deduced MinION consensus sequence showed >99% nucleotide identity compared to the Sanger sequence.

**Fig 1:**
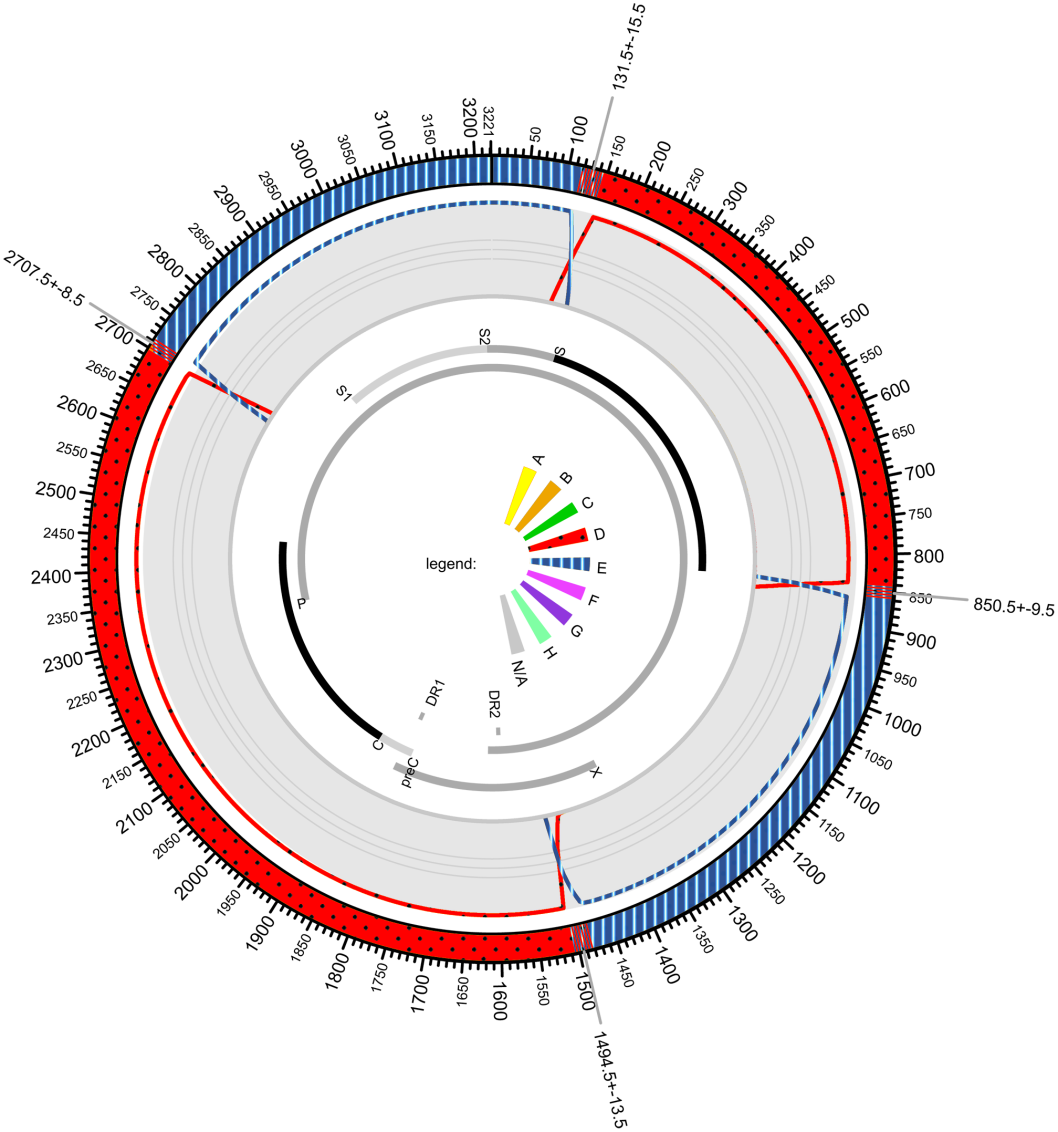
Schematic representation of the recombinant HBV strain B5584. The HBV genome is represented in a circular form and the HBV open reading frames are shown. Breakpoint positions for D (red, dotted)/E (blue, striped) genomic recombination are indicated with arrows and positions are given relative to an HBV genotype A reference sequence (GenBank AM282986).

The 472 nanopore sequences were analyzed with the jpHMM-HBV software (Fig 1). Results identified four genotype D/E recombination breakpoints in the 213 sequences covering ≥ 95% of HBV genome. Similar results were obtained for nine additional sequences covering ≥ 77% of the viral genome. Breakpoints were located at positions 131, 850, 1,494 and 2,707 as indicated in Fig 1 and previously described in the clones sequenced with the Sanger method. The same D/E breakpoints were observed in the genome regions covered by 93 additional partial sequences. The 157 remaining sequences were not interpretable due to too short length and/or high error rate. No parental genotype D and E sequences were detected.

### HBV quasispecies characterization

Analysis of sample B6505 with MinION produced a total of 15,296 sequence reads from 210 of 512 channels from which 2,208 reads (14.4%) categorized as pass reads. When corrected pass reads (n = 135) were considered, 125 reads aligned to the B6505 Sanger sequence (Table 1) and ten non-aligned reads further related to bacterial sequences. The 125 HBV corrected reads had an average length of 2,193 nt (range: 289–3,217 nt) and included 27 reads that cover ≥95% of the HBV genome with an average nucleotide identity of 91.5% (range: 74–98%). The MinION consensus sequence generated was >99% accurate when compared to Sanger sequence.

**Fig 2:**
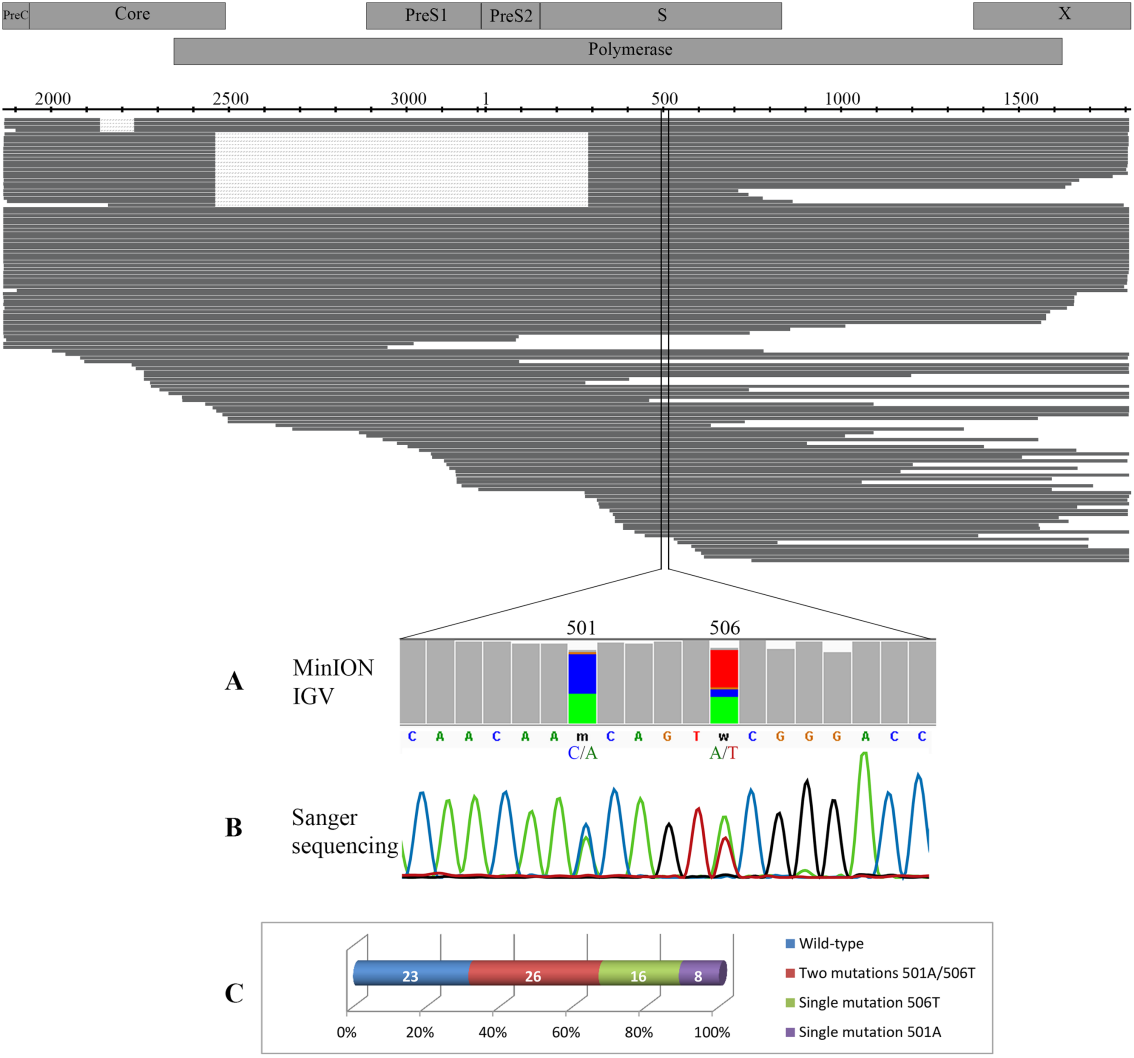
Phasing of the S gene mutations Thr116Asn and Thr118Ser in the HBV genome. Each bar represents one read and the dots indicate a deletion. Both single nucleotide variants were called by MinION (A) and direct Sanger sequencing (B). The number of MinION reads called for each of the single nucleotide variant patterns (C).

Using the long sequencing reads of MinION, we attempted to phase multiple nucleotide polymorphisms across the genome (Fig 2). However, due to the limited number of corrected reads covering the nearly complete HBV genome, it was not possible to phase accurately the overall single nucleotide polymorphism across the viral genome. Thus, the phasing analysis was limited to two variable positions (nt 501A/C and nt 506A/T) of clinical relevance previously identified by the Sanger sequencing. Out of the total 125 HBV reads available, 73 sequences encompassing the two positions qualified for analysis: 23 (31.5%) sequences were “wild-type” (501C/506A), 25 harbored a single substitution (8 [11%] at position 501A and 16 [22%] at position 506T), and 26 (35.5%) carried the two mutations (501A/506T). These results were consistent with the Sanger data. No association between substitutions was observed.

### Identification of deletion HBV variants

Sequencing of amplified products from sample B6260 produced 3,844 reads from 413 of 512 nanopore channels. Only 72 reads (1.9%) categorized as pass reads and 30 corrected reads aligned to the B6260 Sanger sequence (Table 1). These corrected reads were 2,082-nt in length on average (range: 213–3,015 nt) with a mean pairwise nucleotide identity of 89% (range 77–94%). Due to a very low number of reads, the MinION consensus showed only 95% nucleotide identity compared to the Sanger sequence. However, all 30 complete and partial corrected sequences were tested by BLASTn analysis using the NCBI viral database and were matched with HBV genotype D reference sequences.

**Fig 3:**
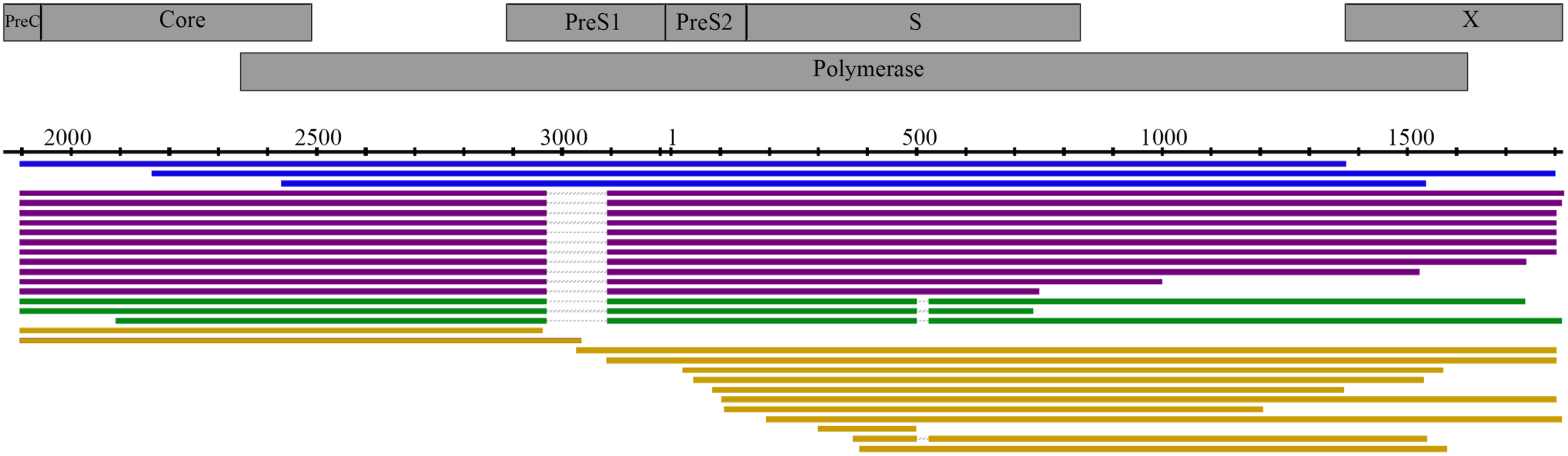
Alignment of nanopore reads of B6260. Complete and partial Nanopore sequence reads were aligned with the corresponding Sanger consensus sequence used as reference. The viral core, surface, polymerase and X proteins are indicated and positions provided according to the reference sequence AM282986. Four different types of Minion reads are shown: nearly complete HBV genome (blue); reads showing a 123-nt deletion (positions 2,968–3,090) in the PreS1 region (purple); reads with the 123-nt and an additional 24-nt deletion (positions 501–524) in the “a determinant” of the S region (green); and unclassified partial reads (yellow).

As shown in Fig 3, three reads corresponded to the nearly complete HBV genotype D2 genome, 11 sequences showed a 123-nt deletion (positions 2,968–3,090) in the PreS1 region, and three sequences showed a 24-nt deletion (positions 501–524) in the “a determinant” of the S region in addition to the 123-nt deletion. The remaining 13 partial sequences were too short to be classified. The two deletions detected by single-molecule sequencing were consistent in size and location with the ones previously identified by cloning and Sanger sequencing.

Similarly, shorter reads were also observed in samples B5584 and B6505 (Table 1). The alignment of two ~1,960 nucleotides long reads from sample B5584 with the full HBV sequence identified a large 1,229-nt deletion in the Pol/PreS1 region that made them potential HBV spliced variants with known splice donor and acceptor sites located at positions 2,471 and 489 [21]. Of note, the recombination breakpoints at positions 850 and 1,494 mentioned above were also found in the spliced sequences. Sequencing an independent PCR product with the Ion Torrent Proton system confirmed the presence of this spliced variant (SAMN08376066). The analysis of sample B6505 reads revealed a diverse population of smaller genomes with 21 reads (range: 1,052–2,167 nt) showing a 1,049-nt deletion potentially associated with a previously described spliced variant with breakpoints at positions 2,447 and 282 [21]. Four other distinct reads (range: 3,065–3,092 nt) with a shorter 96-nt deletion between positions 2,131 and 2,226 were also observed in sample B6505 Core region sample. No cryptic consensus splicing signals (5’- AGGURUGU——YNRCURAC——Yn——YAGG -3’) were observed at 2,131 and 2,226 breakpoints (SAMN08367591).

## Discussion

The aim of the present study was to evaluate the performance of the recently developed Oxford nanopore long-read single-molecule sequencing technology to explore the genetic complexity of a viral infection in a clinical plasma sample using hepatitis B virus (HBV) as a model. MinION was able to generate individual complete 3,200-nt HBV genome sequences, to identify recombinant variants and was particularly efficient in detecting HBV genomes with multiple large in-frame deletions and spliced variants concomitantly with non-deleted parental genomes.

The MinION technology has been applied to bacterial and eukaryotic genome sequencing with reads up to 233 kb [22–25] but its use for viral investigations remains relatively limited. Studies on viruses mainly used the technology for rapid viral identification using both amplicons and shotgun metagenomics sequencing [26–28]. The long nanopore reads also enabled whole genome sequencing of Influenza A virus [29] and Ebola viruses during the 2014–2015 disease outbreak in West Africa [30, 31]. These last studies demonstrated the ability of the MinION^™^ device to be rapidly implemented in complicated clinical situations and to provide rapid viral genome consensus sequencing from field-collected samples allowing rapid diagnostic. However, the performance of this technology for in depth analysis of the viral genetic diversity within a clinical sample remained to be evaluated. HBV is a relevant model due to its well-documented high genetic diversity during the natural course of infection characterized by mixed populations of viruses carrying single nucleotide substitutions and/or deletions/insertions, spliced genomes, and possible inter-strain genetic recombinations [1].

New HBV variants generated by recombination events, including tentative genotypes I and J, have been documented worldwide and intergenotype recombination appears to play a major role in HBV evolutionary history [11]. The method used to obtain complete genome sequences is important because mixed infection with two different genotypes may contribute to artifacts during PCR amplification [2]. Assembly of multiple subgenomic fragment sequences may also lead to the erroneous conclusion that intergenotype recombination occurred [11]. Until now, to differentiate between mixed infection and true recombination, it was advisable to obtain the full-genome sequence from multiple clones of a unique PCR amplified product. In the present study, the use of MinION single-molecule sequencing confirmed the presence of an unique HBV D1/E recombinant variant in sample B5584 (Fig 1) and ruled out the possibility of mixed infection since no corresponding parental genotype D1 and genotype E complete sequences were concomitantly detected. Recombination was documented avoiding the heavy-handed and time-consuming cloning procedure.

In addition, this new approach applying single-molecule sequencing directly to overall amplified products generated in a PCR reaction revealed a higher level of viral genetic complexity in an infected individual than previously estimated by using Sanger sequencing of size-selected amplicons. Indeed, HBV genomes with large deletions were detected in all three samples studied (Table 1). In samples B5584 and B6505, large deletions corresponded to previously described splicing of the HBV genome between well-documented splice donor and acceptor sites located at nucleotides 2,471 and 489 (B5584) and nucleotides 2,447 and 282 (B6505) [21]. These findings are in agreement with a recent study using the PacBio technology [32] and suggest that spliced HBV genomes may be more common, even if present at low levels, in chronically infected individuals than previously estimated [33]. These single-molecule sequencing technologies might be valuable tools to identify known and/or novel spliced variants and to understand the functional role and complex regulation of HBV splicing during the course of HBV infection [32]. Not all observed deletions were associated with potential splicing. Variant genomes carrying multiple in-frame deletions were clearly identified concomitantly to non-deleted parental genomes in samples B6260 (deletion 2,968–3,090 in PreS1 ± deletion 501–524 in S) and B6505 (deletion 2,131–2,226 in core). All these variants would not be able to produce functional L, S or Core proteins. Despite the limited number of sequences analyzed, MinION technology demonstrated its ability to identify co-occurring genetic rearrangements occurring~800 nucleotides apart in sample B6260. The deletion 501–524 was always found associated with the deletion 2,968–3,090 irrespective of the sequencing method used suggesting a possible sequential occurrence. Further investigations with improved sequencing yield are needed to fully estimate the benefit of long read single-molecule sequencing in monitoring HBV genetic evolution pattern.

Overall results obtained with both HBV plasmids and full-genome amplicons showed some technical limitations. First, the number of reads identified as originating from HBV before correction ranged from 72 to 4,190 accounting for 1.9% to 21% of the total reads. As reported by others, the low reads yield (range: 3,844–25,562 reads) was independent of the type and input of DNA but was related to the low proportion of functional nanopores within R7.3 flow-cells (less than 50% out of the 512 channels) [6]. Secondly, a high total error rate of 12% was observed, as previously reported by others irrespective of the DNA material [34]. A similar error rate has been reported with the alternative long-read PacBio system while short-read technologies using Illumina sequencers showed significant lower error rates (< 0.1%) [35, 36]. Therefore, the use of these early versions of the MinION long-read technology for variant calling and phasing mutations proved challenging. However, due to its long-read ability generating full-length viral genome sequences, the MinION showed its potential to improve the characterization of a complex viral population within an infected individual as shown with HBV in the present study. Despite the high error rate mentioned, the pairwise identity of MinION HBV consensus genome was consistent with Sanger sequencing method. Recently, major technological improvements on flow cells and chemistries (R9.0 and R9.4) resulted in increased throughput up to 5–10 Gb of DNA sequencing data comparable to the most commonly used Illumina MiSeq, Ion Torrent PGM and Proton systems, and a ~8% decreased sequence error rates when using 2D R9.0 reads [34, 37]. Such improvements are promising for detection and accurate phasing of mutations across large distances but they still need to be evaluated.

In conclusion, these early MinION versions showed the ability to generate long reads allowing the direct genetic analysis of a complex viral population avoiding the time-consuming cloning procedure. However, it also showed a high sequencing error rate that challenges single-nucleotide resolution and polymorphism calling. The MinION device being under continuous development, further studies are needed to evaluate the potential improvements of this promising technology for viral infection characterization.

## Supporting information

S1 Fig Assessment of MinIon sequence error rate as compared to the time at which the sequence is recorded off the sequencer (A; using all points in the sequencing experiment), and compared to the sequence length (B; using a smooth spline on all points in the sequencing experiment). On the whole, error rates appear to be constant over both sequence length and run time, independently of the type of molecule (2d, template, complement).
(TIF)

S1 Table Assessment of long-read sequence aligners based on largest average alignment length with respect to Sanger sequence references. Long-read sequence aligners that are best fit to the type of data generated by long-read sequencing technologies, among LAST v475 [13], BLASR [17] and LASTZ v 1.02.00 [18] were tested to align nanopore reads to their respective Sanger sequence references including HBV and vector sequences. Considering the largest number of aligned reads, 88.1%-89.1%, with a greater alignment length average, i.e. 3,273–3,388 nt, and a mean alignment identity ranging from 72.8% to 74.6%, we further considered LAST as the best choice in terms of read aligner for subsequent analyses and assessment in this study. Average read length values are rounded to 2 decimal figures; average alignment length values are rounded to the nearest whole number.
(DOCX)
