# Supplementary figures and images for: Early MinION™ nanopore single-molecule sequencing technology enables the characterization of hepatitis B virus genetic complexity in clinical samples
Source: PLoS One. 2018 Mar 22;13(3):e0194366. doi: 10.1371/journal.pone.0194366 (PMC5864009; doi:10.1371/journal.pone.0194366)

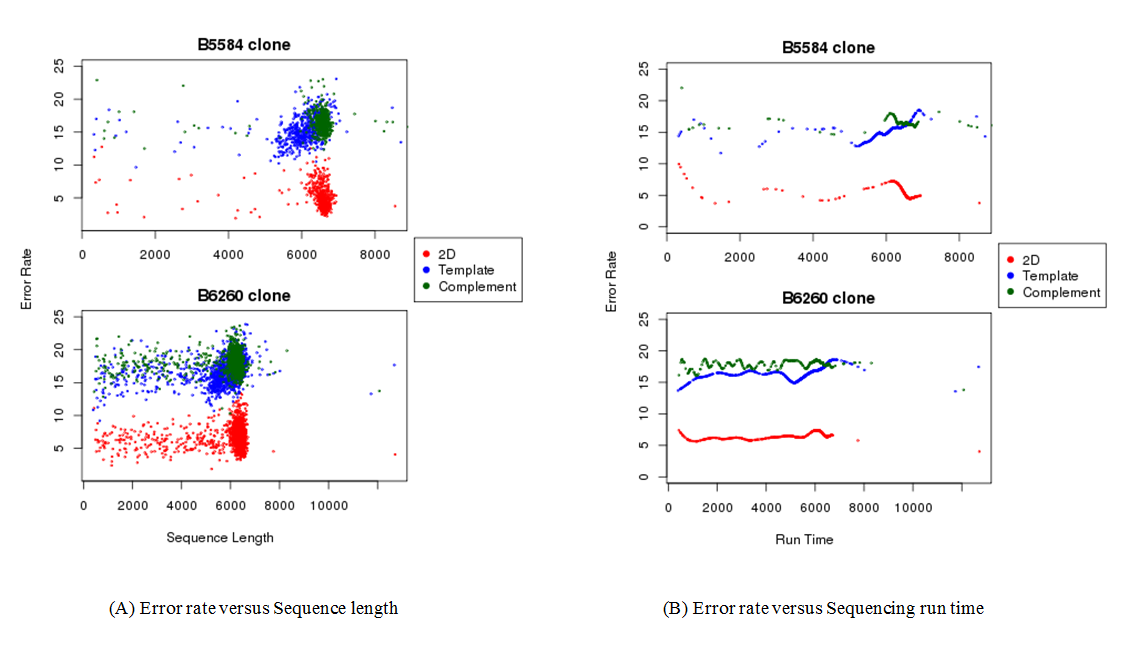

Supplement: S1 Fig — On the whole, error rates appear to be constant over both sequence length and run time, independently of the type of molecule (2d, template, complement). (TIF) [file pone.0194366.s001.tif]
