# Supplementary material for: Early MinION™ nanopore single-molecule sequencing technology enables the characterization of hepatitis B virus genetic complexity in clinical samples
Source: PLoS One. 2018 Mar 22;13(3):e0194366. doi: 10.1371/journal.pone.0194366 (PMC5864009; doi:10.1371/journal.pone.0194366)
Supplement: S1 Table — Long-read sequence aligners that are best fit to the type of data generated by long-read sequencing technologies, among LAST v475 [13], BLASR [17] and LASTZ v 1.02.00 [18] were tested to align nanopore reads to their respective Sanger sequence references including HBV and vector sequences. Considering the largest number of aligned reads, 88.1%-89.1%, with a greater alignment length average, i.e. 3,273–3,388 nt, and a mean alignment identity ranging from 72.8% to 74.6%, we further considered LAST as the best choice in terms of read aligner for subsequent analyses and assessment in this study. Average read length values are rounded to 2 decimal figures; average alignment length values are rounded to the nearest whole number. (DOCX) [file pone.0194366.s002.docx]

**S1 Table. Assessment of long-read sequence aligners, based on largest average alignment length with respect to Sanger sequence references**

|  |  |  |  | **LAST** | | | **BLASR** | | | **LASTZ** | | |
| --- | --- | --- | --- | --- | --- | --- | --- | --- | --- | --- | --- | --- |
|  | Total reads | Pass reads | Mean read length (min-max) | No of mapped reads | Mean Alignt read length (nt) | Mean Alignt Identity | No of mapped reads | Mean Alignt read length (nt) | Mean Alignt Identity | No of mapped reads | Mean Alignt read length (nt) | Mean Alignt Identity |
| **B5584c (6728 nt)** | 7,656 | 1875 | 5,808.4 | 1,670 | 3,388 | 74.6% | 1,357 | 3,202 | 77.25% | 1,374 | 3,416 | 72.45% |
|  |  |  | (226-11,327) | (89.1%)* |  |  | (72.4%)* |  |  | (73.3%)* |  |  |
| **B6260c (6551 nt)** | 25,562 | 4962 | 5,483.1 | 4,372 | 3,273 | 72.8% | 2,908 | 2,923 | 77.75% | 3,262 | 3,253 | 71.4% |
|  |  |  | (391-20,498) | (88.1%)* |  |  | (58.6%)* |  |  | (65.7%)* |  |  |

*, Pourcentage calculated with « Pass » reads; No, number.
